# Supplementary material for: Development of a computer-aided design software for the quantitative evaluation of aesthetic damage
Source: PLoS One. 2019 Dec 18;14(12):e0226322. doi: 10.1371/journal.pone.0226322 (PMC6919621; doi:10.1371/journal.pone.0226322)
Supplement: S1 Code site — 1. (ZIP) [file pone.0226322.s001.zip › [EESC_jr][Projeto]Relatorio_07_21/reports/2/2017.03.30/18:01:12/report.pdf]

Preâmbulo

Na data de 23/04/2016 no horário 10:00, o (a) Perito(a) Professor Doutor Nelson Sakaguti, Cirurgião Dentista, designado(a)para proceder ao exame pericial solicitado em: fabichona luiz, do sexo Não informado, nascido(a) em 23/02/1980, ocupação marketing, portador(a) do RG 987 ssp, cujo endereço rua waldema rferreira 23, passa a expor a avaliação de dano estético corporal.

Exame objetivo da lesão

A lesão ora objeto de análise teve como natureza Acidente de trânsito ocorrido em 21/02/2017. O prejuízo estético consiste em lesão cicatricial consolidada na região: Rosto, Nariz, Boca no lado direito.

Trata-se de cicatriz Hipertrófica de tamanho/frequência Única: Retilínea de orientação Horizontal de pigmentação Normocrômica medindo 4 centímetro(s) na horizontal, 3 centímetros na vertical e 3 milímetros de Altura. De contornos Irregulares. Perceptivel em 50 centímetros de distância nas relações íntimas. Perceptivel em 3 metros nas relações sociais. A lesão tem repercução na vida diária afetiva, familiar, social, laboral conforme fotos anteriores e atuais..

Conclui-se que classificada segundo os parâmetros de dano estético AIPE - Análise do Impacto do Prejuízo Estético (escala de seis níveis e pontuação entre 0 e 50 pontos), como sendo dano estético relativo à área 1, a lesão é caracterizada por 23 pontos e grau de prejuízo estético: Leve.

Escore final: 23;
Grau de prejuízo: Leve.

FOUSP, 23/04/2016

____

Professor Doutor Nelson Sakaguti
